# Effect of Solid-State Fermented Brown Rice Extracts on 3T3-L1 Adipocyte Differentiation

**DOI:** 10.4014/jmb.2301.01041

**Published:** 2023-04-17

**Authors:** Su Bin Ji, Chae Hun Ra

**Affiliations:** Department of Food Science and Biotechnology, College of Engineering, Global K-Food Research Center, Hankyong National University, Anseong-Si 17579, Republic of Korea

**Keywords:** Solid-state fermentation, fermented brown rice, β-glucan, α-glucosidase inhibition, 3T3-L1 adipocytes

## Abstract

*Aspergillus oryzae* KCCM 11372 was used to enhance the production of β-glucan using humidity control strategies. Under conditions of 60% humidity, solid-state fermentation (SSF) increased the yields of enzymes (amylase and protease), fungal biomass (ergosterol), and β-glucan. The maximum concentrations obtained were 14800.58 U/g at 72 h, 1068.14 U/g at 120 h, 1.42 mg/g at 72 h, and 12.0% (w/w) at 72 h, respectively. Moreover, the β-glucan containing fermented brown rice (β-glucan-FBR) extracts at concentrations of 25–300 μg/ml was considered noncytotoxic to 3T3-L1 preadipocytes. We then studied the inhibitory effects of the extracts on fat droplet formation in 3T3-L1 cells. As a result, 300 μg/ml of β-glucan-FBR extracts showed a high inhibition of 38.88% in lipid accumulation. Further, these extracts inhibited adipogenesis in the 3T3-L1 adipocytes by decreasing the expression of C/EBPα, PPARγ, aP2, and GLUT4 genes.

## Introduction

Solid-state fermentation (SSF) processes have the benefit of accommodating substantial volumes of biomass, are simple to scale up, have low impact on the environment, and do not consume high amounts of water [1]. Different types of agri-food industries have used SSF to produce enzymes, bioactive compounds, high-quality animal feed supplements, and flavors [2] In this study, we aimed to produce β-glucan using brown rice as the source material in SSF.

Brown rice (*Oryza sativa* L.), which is paddy rice that has been hulled, consists of a bran layer, an embryo, and an endosperm. Compared with polished white rice, brown rice (BR) is covered with fine hairs and contains large amounts of nutrients, such as soluble dietary fiber, proteins, fats, vitamins, minerals, and phytochemicals [3]. In a previous study, we sought to determine whether SSF of brown rice using *A. oryzae* KCCM 12698 enhanced the production of enzymes and β-glucan [4]. Based on the optimal medium composition, we employed *Aspergillus oryzae* KCCM 11372 to optimize β-glucan production using humidity control strategies. β-glucans play notable and beneficial roles in insulin resistance, dyslipidemia, hypertension, and weight loss [5, 6, 7]. Kanagasabapathy *et al*. [8] demonstrated the ability of mushroom β-glucan to activate the AMP-activated protein kinase signaling pathway and induce lipolysis in differentiated 3T3-L1 adipocytes.

Obesity, which is characterized by increased adipose tissue mass, is a chronic metabolic disorder caused by an imbalance between food intake and energy homeostasis [9, 10]. The 3T3-L1 cell line is one of the most well-characterized and reliable models for studying adipogenesis [10]. As a process, adipogenesis is controlled by a large number of transcription factors, including CCAAT/enhancer binding protein (C/EBP) family members (especially C/EBPα) and peroxisome proliferator-activated receptor-γ (PPARγ) [10, 11]. Similarly, other transcription factors are key players in adipogenesis: Kruppel-like factors (KLFs), cyclic AMP response element binding protein (CREB), sterol regulatory binding protein-1 (SREBP1), adipocyte fatty acid binding protein (aP2), glucose transporter 4 (GLUT4), and early β-cell factor 1 (EBF1) [11-13]. Therefore, we investigated whether the β-glucan containing fermented brown rice (β-glucan-FBR) extracts modulates fat accumulation and adipogenesis in cultured 3T3-L1 adipocytes.

Our objective in this study was to optimize the humidity conditions for β-glucan production by SSF processes and to demonstrate their potential anti-obesity effects using 3T3-L1 adipocytes. The principal regulators of adipogenesis (C/EBPα and PPARγ) and PPARγ target genes (aP2 and GLUT4) were measured by quantitative real-time PCR.

## Materials and Methods

### Solid-State Fermentation of Brown Rice

One milliliter of spore suspension (1 × 10^8^ spores/ml) was inoculated into a 250-ml Erlenmeyer flask containing 100 ml of modified synthetic medium (30 g/l brown rice powder, 30 g/l rice bran, 10 g/l soytone, and 3 g/l ascorbic acid). Nutrient supplements of 5 g/l K_2_HPO_4_ and 0.25 g/l MgSO_4_ were added to the modified synthetic medium and mixed thoroughly before inoculation. The culture was incubated for 48 h at 150 rpm (30°C) on a rotary shaker and used as the seed culture.

Brown rice (Korea) was pretreated by soaking 100 g (of rice) in 200 ml of water for 2 h in rectangular trays, which were then covered with aluminum foil, and autoclaved at 121°C for 60 min. The nutrient supplements of freshly modified synthetic medium (10 ml) were added to the rectangular trays and mixed thoroughly before inoculation. *A. oryzae* KCCM 11372 was cultivated by SSF using brown rice as a substrate and contained in 500-ml stainless steel rectangular trays with a 200-ml working volume. SSF was performed at 30°C for 5 days and at 50–80% relative humidity using a constant temperature and humidity incubator (BH-120CA/B; Neuronfit Co., Ltd., Korea). Samples were collected periodically, lyophilized using a freeze dryer, and stored at 20°C before the measurement of amylase, protease, fungal biomass (ergosterol), and β-glucan content. The enzyme and fungal biomass (ergosterol) contents were determined in accordance with previously described procedures [4].

The dried, ground non-fermented or fermented brown rice (3 g) was extracted with 100 ml of 80% (v/v) ethanol at 60°C for 2 h. The extracted solution was filtered using filter paper (8 μm; Whatman, UK) and concentrated at 80°C in a rotatory vacuum evaporator (EYELA N-1000, Riakikai Co., Ltd., Japan). The extracts were then used in subsequent experiments.

### Determination of β-Glucan Content

The β-glucan content of brown rice was carried out using the β-Glucan Assay Kit (Mixed Linkage; Ireland). The β-glucan content was determined according to the procedure described by Yoo *et al*. [14]. Briefly, pretreated extracts (0.1 g) were suspended and hydrated in a buffer solution. The mixture was then incubated with purified lichenase and filtered. Aliquots (0.1 ml) of the filtrate were then hydrolyzed to completion using purified β-glucosidase. The amount of D-glucose produced was determined using a glucose oxidase/peroxidase reagent.

### 3T3-L1 Cell Culture and Adipocyte Differentiation

3T3-L1 preadipocytes were obtained from the Korean Cell Line Bank (KCLB, Korea). The pre-adipocyte differentiation assay was performed using the modified method proposed by Lim *et al*. [15]. The cells were grown in DMEM containing 10% (v/v) BCS and 1% (v/v) penicillin/streptomycin at 37°C in a humidified atmosphere of 95% air and 5% CO_2_ using a constant temperature & humidity incubator (BH-120CA/B; Neuronfit Co., Ltd., Korea). Fresh medium was provided to the cells every 2–3 days until they reached approximately 80% confluence. Then, 3T3-L1 preadipocytes were seeded in 12-well plates at a density of 5 × 10^4^ cells/well to differentiate preadipocytes into adipocytes. Cells were differentiated in 2 days in a differentiation medium (DMEM supplemented with 10% FBS, 1 μM dexamethasone (DEX), 0.5 mM isobutyl-1-methylxanthine (IBMX), and 10 μg/ml insulin). On day 2, the medium was replaced with a maturation medium (DMEM, 10% FBS, and 10 μg/ml insulin), which was changed every 2 days until day 8.

### α-Glucosidase Inhibitory Assay

The α-glucosidase inhibitory activity was performed with the method described by Ye *et al*. [16]. Briefly, 50 μl of the β-glucan containing brown rice extracts (dissolved in 50 mM potassium phosphate buffer (pH 6.8)) at various concentrations was mixed with 50 μl of α-glucosidase (dissolved in 50 mM potassium phosphate buffer). After 30 min of incubation at 37°C, 100 μl of 3 mM pNPG was added to the mixture and incubated for another 30 min at 37°C. The reaction was stopped by adding 750 μl of 0.1 M Na_2_CO_3_ solution. The absorbance was measured at 405 nm using a microplate reader (Multiskan FC, Thermo Fisher Scientific, USA). The β-glucan (positive control) with IC_50_ values was previously reported to be 284.50 μg/ml [17]. The inhibitory effects of the tested compounds were calculated at the same concentration as the IC_50_ value. Acarbose (0.28 mg/ml) and phosphate buffer were used as positive and negative controls, respectively. The percentage of α-glucosidase inhibition was calculated using Eq. (1):

$$\text{α-Glucosidase inhibition = }\left[\text{1-}\left(\frac{\text{(Absorbance of sample - Absorbance of blank)}}{\text{(Absorbance of control)}}\right)\right]\times 100.$$ (1)

### MTT Assay

The MTT assay was carried out to investigate the viability of 3T3-L1 preadipocytes according to the modified method of Vaidya *et al*. [18]. First, 3T3-L1 cells were seeded into a 96-well plate at a density of 1 × 10^4^ cells/well in DMEM and allowed to attach for 24 h. Cells were then treated with different concentrations (25–300 μg/ml) of fermented brown rice (FBR) extracts for 48 h at 37°C in 5% CO_2_ and the untreated cells served as a control. Next, 10 μl of MTT solution (5 mg/ml) in PBS (pH 7.4) was added to each well and incubated in the dark for 2 h at 37°C. The MTT reagent was removed and the formed formazan crystals were dissolved in DMSO (200 μl/well). The absorbance of each well was measured at 570 nm wavelength using a microplate reader. The cell viability (%) can be expressed by Eq. (2):

$$\text{Cell viability = }\left(\frac{\text{(Absorbance of sample-Absorbance of blank)}}{(\text{Absorbance of control-Absorbance of blank)}}\right)\times 100.$$ (2)

### Oil Red O Staining

Cytoplasmic lipid droplets were measured using Oil Red O as previously described by Ramírez-Zacarías *et al*.[19]. The cells were washed thrice with PBS (pH 7.4) and fixed in 10% (v/v) formalin at room temperature for 1 h. After the removal of 10% formalin, the wells were washed with 60% isopropyl alcohol for 5 min and then washed exhaustively with PBS. Thereafter, the wells were allowed to dry completely for 20 min before the addition of filtered Oil Red O solution. The stained cytoplasmic lipid droplets were visualized and photographed under a microscope. For quantification of Oil Red O content, the cells were rinsed thrice with distilled H_2_O to remove background staining. The stained oil droplets were extracted with 100% isopropanol for 10 min. The extracted dye was immediately removed and its absorbance was measured at 492 nm using a microplate reader (Multiskan FC, Thermo Fisher Scientific, USA).

$$\text{Lipid accumulation = }\left(\frac{\left(\text{Absorbance of sample}\right)}{\left(\text{Absorbance of control}\right)}\right)\times 100.$$ (3)

### Quantitative Real-Time PCR

Total RNA from 3T3-L1 cells was extracted using an RNA Extraction Kit (MGmed, Korea), following the manufacturer’s instructions. Newly synthesized cDNA from total RNA was amplified using specific primers and (SYBR Gerrn), Dyne One-step RT qPCR kit (Dyne Bio Inc, Korea). Quantification of mRNA expression in 3T3-L1 cells treated with fermented brown rice extract was performed using a CRX Connect Real-Time PCR system (Bio-Rad). The target gene sequences were obtained from NCBI. The primers used in this study were designed using the NCBI BLAST website, and are listed in Table 1. GAPDH was used as the internal standard gene. The RT-qPCR conditions were as follows: reverse transcription at 42°C for 30 min, initial denaturation at 95°C for 5 min, denaturation at 95°C for 30 s, annealing at 55°C for 30 s, and extension at 72°C for 30 s. This step was repeated 40 times. The relative amount of each gene was calculated using the 2^ΔΔCt^ method [20].

### Statistical Analysis

Each experiment was performed in triplicate. Statistical significance was evaluated by one-way analysis of variance (ANOVA) and Duncan’s multiple range test (*p* < 0.05) using SPSS version 23 (SPSS, USA).

## Results and Discussion

### Solid-State Fermentation with Various Humidity Conditions

**Fig. 1:**
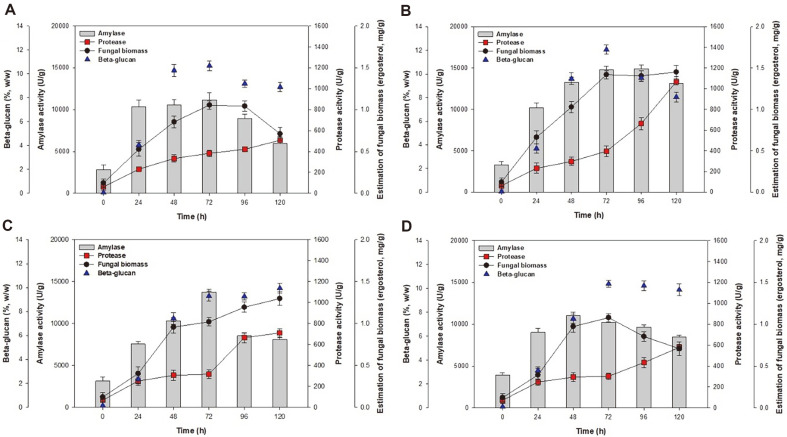
Optimization of enzymes and β-glucan productions for SSF by changing the humidity conditions. (**A**) 50%, (**B**) 60%, (**C**) 70%, and (**D**) 80%. The modified synthetic medium composition was 30 g/l brown rice, 30 g/l rice bran, 20 g/l soytone, 3 g/l ascorbic acid, and nutrient supplements. The initial pH was 5.5, the temperature was 30°C, and fermentation of *A. oryzae* KCCM 11372 was conducted for 120 h.

As shown in Fig. 1, SSF under various humidity conditions from 50 (Fig. 1A), 60 (Fig. 1B), 70 (Fig. 1C), and 80%(Fig. 1D) was performed using 500-ml rectangular trays with a working volume of 200 ml. Fig. 1A shows that the growth of fungal biomass at 50% humidity grew into a stationary phase at 72 h and produced fungal biomass of 1.05 mg/g. When the fungal biomass decreased from 72 to 120 h, the production of amylase and β-glucan ceased. The maximum levels of amylase, protease, and β-glucan production were obtained with 11132.65 U/g at 72 h, 382.64 U/g at 120 h, and 10.67% (w/w) at 72 h, respectively. These results indicated that there was a significant humidity gradient (data not shown). Similar results were reported by He *et al*. [21]; low moisture content inhibits microbial growth and enzyme production and limits nutritional transfer. Furthermore, lower moisture content was combined with the best sporulation conditions [22].

As shown in Fig. 1B, the effect of 60% humidity indicated that each of the products rapidly increased during fermentation. The maximum concentrations of enzymes (amylase and protease) and the fungal biomass (ergosterol) were 14800.58 U/g at 72 h, 1068.14 U/g at 120 h, and 1.42 mg/g at 72 h, respectively. The highest β-glucan content was 12.0% (w/w) at 72 h.

The results clearly indicated that 60% humidity in the SSF process can be a suitable condition for the production of enzymes, fungal biomass (ergosterol), and β-glucan compared to those obtained with 50% humidity (Fig. 1A). For this reason, proper humidity condition plays a key role in the moisture content during SSF, which affects the physicochemical properties [23]. Thus, 60% was selected as the optimal humidity condition for this study.

Figs. 1C and 1D show the production of enzymes, fungal biomass (ergosterol), and β-glucan under 70 and 80%humidity conditions in brown rice in SSF. The production of enzymes, fungal biomass (ergosterol), and β-glucan was lower than that at 60% humidity (Fig. 1B). As shown in Fig. 1C, the maximum production of enzymes (amylase and protease) and fungal biomass (ergosterol) were 13717.84 U/g at 72 h, 710.61 U/g at 120 h, and 1.29 mg/g at 120 h, respectively. The highest β-glucan content was obtained at 9.9% (w/w) for 120 h. Similarly, Fig. 1D shows that the best enzyme (amylase and protease), fungal biomass, and β-glucan yields of 11054.95 U/g at 48 h, 578.20 U/g at 120 h, 1.07 mg/g at 72 h, and 10.3% (w/w) at 72 h, respectively, were obtained at 80% humidity. These results indicate that 70–80% humidity did not lead to a significant increase in product yield. The high moisture content makes the substrate wet, making it non-conducive to fungal biomass formation. Similar results were obtained previously for humidity control strategies for solid-state fermentation, which showed that wet fungus induces the formation of a water film on the substrate surface and leaves the scope for limited ventilation [21].

### α-Glucosidase Inhibitory Activity of Brown Rice Extract

**Fig. 2:**
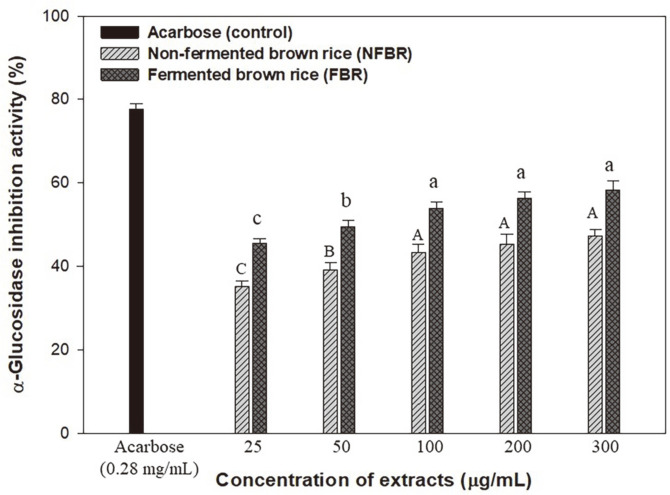
Effect of NFBR extracts and β-glucan containing FBR extracts on α-glucosidase inhibitory activity. The tested compounds were calculated based on the same concentration of β-glucan as IC_50_ value (0.28 mg/ml). Different capital and small letters indicate significant differences (*p* < 0.05, Duncan’s test).

β-Glucan is abundant in the cell walls of cereal grains, mushrooms, and yeasts. Generally, cereal grains contain mainly (1,3/1,4)-β-glucans, whereas mushrooms contain (1,3/1,6)-β-glucans [24]. Crude ethanolic extracts of non-fermented brown rice (NFBR) and β-glucan-FBR were evaluated for their α-glucosidase inhibitory activity, which ranged from 25 to 300 μg/ml, as shown in Fig. 2. The β-glucan containing the NFBR extract was close to zero under the experimental conditions (data not shown). Thus, the inhibitory effects of NFBR extract compounds were evaluated with the same dilution conditions of β-glucan-FBR extract and compared to acarbose (0.28 mg/ml) as a positive control. Fig. 2 shows that the percentage inhibition of β-glucan-FBR extracts was statistically significant, with higher inhibition values compared to those with NFBR extract. At the lowest concentration of 25 μg/ml, the inhibition percentages for NFBR and FBR extracts were 35.16 and 45.48%, respectively, while at the highest concentration of 300 μg/ml, the inhibition percentages were 47.19% for NFBR and 58.39% for FBR. According to the estimated 50% inhibitory concentration (IC_50_) value, FBR had the lowest IC_50_ (92.93 μg/ml), when compared to NFBR (317.86 μg/ml) and acarbose (183.26 μg/ml). Generally, a lower IC_50_ value indicates better inhibitory activity, as it suggests that fewer samples are required to inhibit the enzyme [25]. Thus, 100 μg/ml of β-glucan content (FBR) is expected to be well-suited for α-glucosidase inhibitors (IC_50_) and can play a role in controlling blood glucose levels.

### Adipocyte Cell Viability

**Fig. 3:**
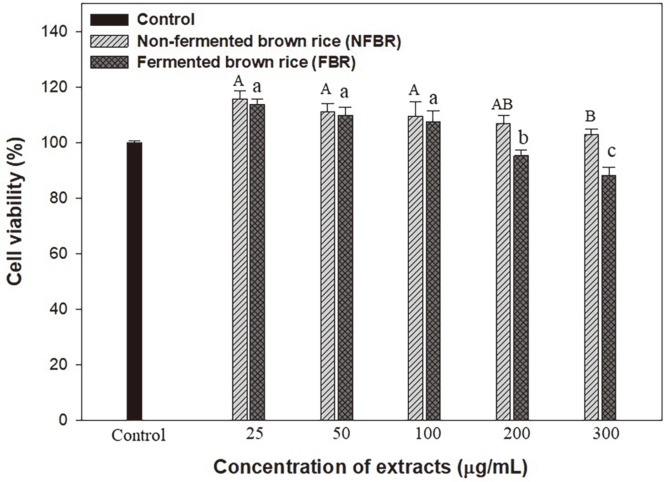
Cell viabilities of 3T3-L1 cells after pretreatment with NFBR extracts and β-glucan containing FBR extracts. Cultures in basal medium served as control. Different capital and small letters indicate significant differences (*p* < 0.05, Duncan’s test).

MTT assay was performed to assess the viability of 3T3-L1 preadipocytes treated with various concentrations of NFBR extracts and the β-glucan-FBR extracts, as shown in Fig. 3. The cell viabilities ranged from 115.69 to 88.04%after 48 h of incubation with NFBR extracts and the β-glucan-FBR extracts. The cell viabilities of the FBR extracts at high concentrations (200–300 μg/ml) were significantly lower than those of the control and NFBR extracts. Pursuant to ISO 10993-5, percentages of cell viability above 80% are considered non-cytotoxic compared with those of untreated cells (control) [26]. These results indicated that all extracts at different concentrations had the highest concentration of 300 μg/ml, which was significantly higher than 80%. Similar results were reported by Kanagasabapathy *et al*. [8]. The β-glucan-rich polysaccharides (GE) exerted a dose-dependent increase in the proliferation of preadipocytes, suggesting that GE was not cytotoxic to 3T3-L1 adipocytes. Therefore, NFBR extracts and the β-glucan-FBR extracts at concentrations of 25–300 μg/ml were considered non-cytotoxic to 3T3-L1 preadipocytes.

### Oil Red O Staining for Lipid Content

**Fig. 4:**
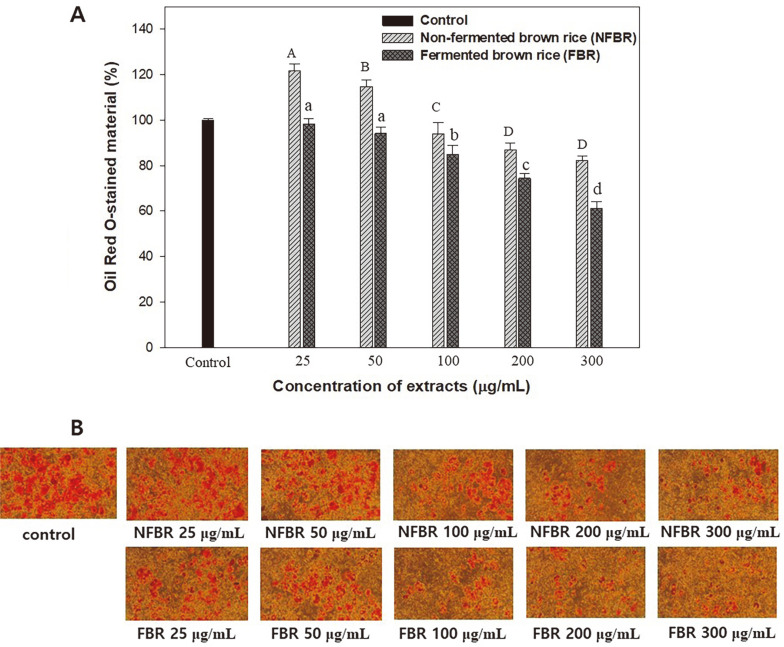
Relative lipid content quantified using Oil Red O staining in 3T3-L1 adipocytes (A) and light micrographs of differentiating adipocytes treated with NFBR extracts and β-glucan containing FBR extracts. Different capital and small letters indicate significant differences (*p* < 0.05, Duncan’s test).

To determine the inhibitory effects of NFBR extracts and the β-glucan-FBR extracts on fat droplet formation in 3T3-L1 cells, we employed the quantification method of Oil Red O staining. As shown in Fig. 4A, the lipid accumulation of β-glucan containing FBR extracts at high concentrations (100–300 μg/ml) is significantly lower than that of the control, but the lipid accumulation of 3T3-L1 preadipocytes treated with NFBR extracts does not show significant differences compared to the control. Interestingly, lipid accumulation in 3T3-L1 preadipocytes treated with NFBR extracts at 25–50 μg/ml was higher than that in the control. A possible explanation for these results is that lipophilic constituents, such as the unsaponifiable fraction (phytosterols, triterpene alcohols, 4-methyl-sterols), less polar components (squalene or tocotrienols and α-tocopherol), and γ-oryzanol, cannot be extracted with polar extractants. On the other hand, hydrophilic constituents, such as polyphenols, GABA, and reducing sugars, may be extracted [27, 28]. These active compounds in the extract may have contributed to this observation. The β-glucan-FBR extracts at a concentration of 300 μg/ml showed a high inhibition of lipid accumulation by 38.88%. As shown in Figs. 4A and 4B, although NFBR extracts are able to slightly reduce lipid accumulation in adipocytes, the β-glucan-FBR extracts provide better inhibition. Similar results were observed for germinated brown rice extracts on pancreatic lipase, adipogenesis, and lipolysis in 3T3-L1 adipocytes. The water and ethyl acetate extracts at a concentration of 300 μg/ml exhibited the lowest oil red O staining with 38.45–34.83% [15].

### Effects of the Transcript Levels on Adipogenesis Genes

**Fig. 5:**
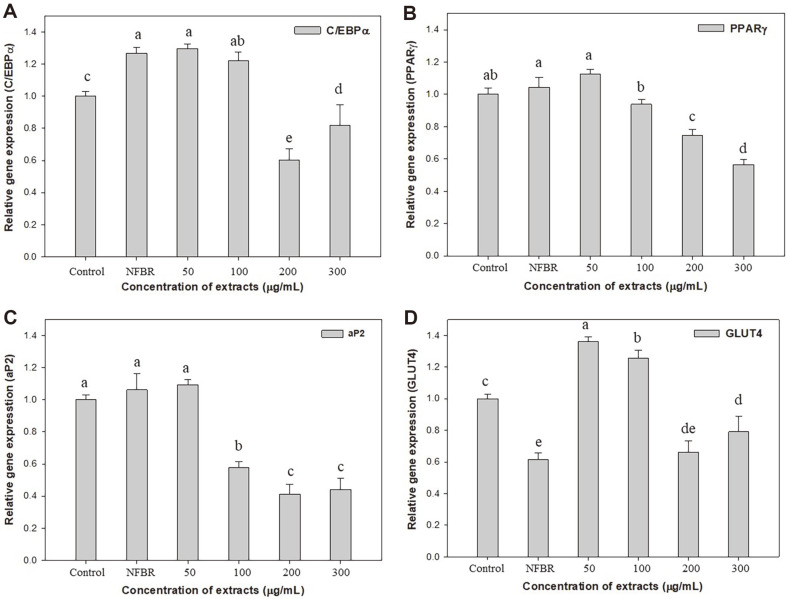
Expression levels of C/EBPα, PPARγ, aP2, and GLUT4 in 3T3-L1 cells. Cultures in basal medium and NFBR extract of 100 μg/ml served as the control. Different letters indicate significant differences (*p* < 0.05, Duncan’s test).

Adipogenesis is inhibited by several molecules and compounds. Among them, β-glucan inhibits this process. To further investigate the suppressive effects of the β-glucan-FBR extracts on lipid accumulation, the mRNA levels of C/EBPα, PPARγ, aP2, and GLUT4 in 3T3-L1 cells were determined using RT–qPCR, as shown in Fig. 5. The collaboration of PPARγ and C/EBPα is critical for the expression of enzymes involved in triglyceride synthesis in the late stage or terminal differentiation [29]. As shown in Figs. 5A and 5B, the mRNA expression levels of C/EBPα and PPARγ are reduced to 39.6% with 200 μg/ml FBR extract and 43.5% with 300 μg/ml FBR extract compared to those of the control. These results indicate that the β-glucan-FBR extracts significantly downregulate the mRNA expression levels of C/EBPα and PPARγ. Furthermore, the suppressive effects of the β-glucan-FBR extracts are stronger than those of the NFBR extracts. These results are consistent with a previous report that high-molecular-weight barley β-glucan inhibits lipid accumulation in 3T3-L1 adipocytes by downregulating the mRNAs of C/EBPα and PPARγ [30].

As shown in Figs. 5C and 5D, the mRNA expression levels of aP2 and GLUT4 are reported to be late markers of adipocyte differentiation [31]. aP2 is an adipocyte-specific gene involved in fatty acid metabolism that can be activated by C/EBPα and PPARγ [31]. GLUT4 also creates and maintains the adipocyte phenotype and is a key regulator of glucose homeostasis in mammals [32]. Fig. 5C shows that the mRNA expression of aP2 genes is dose-dependently downregulated by β-glucan content in FBR extracts. A further increase in the β-glucan-FBR extracts over 100 μg/ml results in a decrease in the mRNA expression levels of aP2 genes, such as 42.3 (200 μg/ml) and 56.0% (300 μg/ml) for 3T3-L1 adipocyte differentiation. As shown in Fig. 5D, GLUT4 gene expression levels at 50 and 100 μg/ml are higher than those in the control. This indicated that low concentrations of FBR extracts did not significantly suppress adipogenesis transcription factors, such as C/EBPα and PPARγ. This may be due to the induction of PPARγ in non-adipogenic fibroblasts being sufficient to promote their differentiation into mature adipocytes by increasing the expression of GLUT4 [32]. Similarly, bacterial β-glucan from *Aureobasidium* sp. inhibited adipogenesis in 3T3-L1 adipocytes by decreasing the expression of PPARγ, acetyl-CoA carboxylase (ACC), and FABP4 (aP2) genes [33]. Based on the quantitative RT-PCR results, the β-glucan-FBR extracts strongly inhibited adipogenesis in 3T3-L1 adipocytes.

On the other hand, many kinds of biologically active components in *A. oryzae*-mediated fermentation products of brown rice have the potential to improve diseased conditions and maintain human health [34]. Therefore, the process of fermenting brown rice with bacterial or fungal strains can beneficially alter the bioactivity.

## Conclusion

Solid-state fermentation (SSF) was performed at 30°C and 50–80% relative humidity using 500-ml stainless steel rectangular trays with a 200-ml working volume. The SSF process allows efficient production of fungal biomass, enzymes, and β-glucan from brown rice. Therefore, we investigated whether the β-glucan containing fermented brown rice (FBR) extracts modulates fat accumulation and adipogenesis in cultured 3T3-L1 adipocytes. Furthermore, mRNA expression levels of the principal regulators of adipogenesis, such as C/EBPα, PPARγ, aP2, and GLUT4, were measured using RT-qPCR. The results obtained from these studies can contribute to the development of the β-glucan containing FBR extract-based functional foods for anti-obesity effects.

## Figures and Tables

**Table 1:** Primer sequence used for real-time qPCR.

Gene	Primer Sequence	
	Forward primer (5' → 3')	Reverse primer (5' → 3')
PPARγ	AGGCTTCCACTATGGAGTTC	CCAACAGCTTCTCCTTCTC
C/EBPα	CAAGAACAGCAACGAGTACC	TTGACCAAGGAGCTCTCA
aP2	GGATTTGGTCACCATCCGGT	TTCACCTTCCTGTCGTCTGC
GLUT4	CCCCCGATACCTCTACATCATC	GCATCAGACACATCAGCCCAG
GAPDH	AACTTTGGCATTGTGGAAGG	ACACATTGGGGGTAGGAACA
